# Publication of Results of Registered Trials With Published Study Protocols, 2011-2022

**DOI:** 10.1001/jamanetworkopen.2023.50688

**Published:** 2024-01-08

**Authors:** Colby J. Vorland, Andrew W. Brown, Halil Kilicoglu, Xiangji Ying, Evan Mayo-Wilson

**Affiliations:** 1 Department of Epidemiology and Biostatistics, Indiana University School of Public Health–Bloomington; 2 Department of Biostatistics, University of Arkansas for Medical Sciences, Little Rock; 3 Arkansas Children’s Research Institute, Little Rock; 4 School of Information Sciences, University of Illinois at Urbana-Champaign; 5 Department of Epidemiology, Johns Hopkins Bloomberg School of Public Health, Baltimore, Maryland; 6 Department of Epidemiology, University of North Carolina Gillings School of Global Public Health, Chapel Hill

## Abstract

**Importance:**

Publishing study protocols might reduce research waste because of unclear methods or incomplete reporting; on the other hand, there might be few additional benefits of publishing protocols for registered trials that are never completed or published. No study has investigated the proportion of published protocols associated with published results.

**Objective:**

To estimate the proportion of published trial protocols for which there are not associated published results.

**Design, Setting, and Participants:**

This cross-sectional study used stratified random sampling to identify registered clinical trials with protocols published between January 2011 and August 2022 and indexed in PubMed Central. Ongoing studies and those within 1 year of the primary completion date on ClinicalTrials.gov were excluded. Published results were sought from August 2022 to March 2023 by searching ClinicalTrials.gov, emailing authors, and using an automated tool, as well as through incidental discovery.

**Main Outcomes and Measures:**

The primary outcome was a weighted estimate of the proportion of registered trials with published protocols that also had published main results. The proportion of trials with unpublished results was estimated using a weighted mean.

**Results:**

From 1500 citations that were screened, 308 clinical trial protocols were included, and it was found that 87 trials had not published their main results. Most included trials were investigator-initiated evaluations of nonregulated products. When published, results appeared a mean (SD) of 3.4 (2.0) years after protocol publications. With the use of a weighted mean, an estimated 4754 (95% CI, 4296-5226) eligible clinical trial protocols were published and indexed in PubMed Central between 2011 and 2022. In the weighted analysis, 1708 of those protocols (36%; 95% CI, 31%-41%) were not associated with publication of main results. In a sensitivity analysis excluding protocols published after 2019, an estimated 25% (95% CI, 20%-30%) of 3670 (95% CI, 3310-4032) protocol publications were not associated with publication of main results.

**Conclusions and Relevance:**

This cross-sectional study of clinical trial protocols published on PubMed Central between 2011 and 2022 suggests that many protocols were not associated with subsequent publication of results. The overall benefits of publishing study protocols might outweigh the research waste caused by unnecessary protocol publications.

## Key Points

**Question:**

What proportion of registered clinical trials with published study protocols do not publish their results?

**Findings:**

In this cross-sectional study of 308 clinical trial protocols published on PubMed Central between 2011 and 2022, weighted analysis found that approximately 36% of trials with published protocols did not publish their main results.

**Meaning:**

This study suggests that many published trial protocols are not associated with published trial results, but the overall benefits of publishing protocols might outweigh the research waste caused by unnecessary protocol publications.

## Teaser

This cross-sectional study estimates the proportion of published trial protocols for which there are not associated published results.

## Introduction

Clinical trials inform regulatory decisions, clinical guidelines, and practice. To prevent selective nonreporting of trials and trial results, the International Committee of Medical Journal Editors requires that trials be registered prospectively to be considered for publication.^1,2,3^ Registration (eg, on ClinicalTrials.gov) promotes transparency; however, registers include only minimal structured data about trials.^4,5,6^ By comparison, study protocols often contain more information about trial methods, interventions, and other details that can help readers interpret and apply trial results.

Publishing trial protocols aims to increase transparency and reduce research waste arising from unreplicable and unusable research.^7^ Protocols for trials of many regulated products and protocols for trials funded by the National Institutes of Health must be posted when study results are posted on ClinicalTrials.gov.^8,9,10,11^ Some journals (eg, *JAMA*, the *New England Journal of Medicine*, *BMJ*, and *Annals of Internal Medicine*) also publish trial protocols as supplements to reports of results.^12^ We are not aware of any funders or journals that require prospective protocol publication in a journal or prospective posting on a preprint server.

Publishing or posting protocols prospectively might help reduce research waste because of unclear methods or incomplete reporting of results. On the other hand, publishing or posting protocols prospectively could contribute to unblinding, deviations from intended interventions, or biased outcomes assessment.^13^ Moreover, there might be few additional benefits of publishing protocols in journals for registered trials that are never completed or published. In this way, publishing study protocols might contribute to research waste. For example, *Trials* has offered to publish trial protocols since 2006; protocols might undergo external review or, for protocols that have been reviewed to obtain grant funding and registered, expedited review by 1 editorial board member.^14^ Open access journals charge thousands of dollars to publish a trial protocol (eAppendix 1 and eTable 1 in Supplement 1), and these monetary costs do not include unpaid time spent drafting, reviewing, editing, and revising protocols. For trials that are never performed or never published, the money and time spent publishing study protocols might be wasted.

Some studies have investigated whether registered trials are associated with published results.^15,16,17,18,19,20,21,22^ For example, studies have estimated that 30% to 64% of trials registered on ClinicalTrials.gov are not associated with published results.^16,17,19,22,23^ Others have investigated retrospectively whether completed trials were registered and had available protocols.^12,15,24,25^ Broadly, these studies suggest that prospective registration of clinical trials is improving over time. They also indicate that protocols are available for many clinical trials; however, prospective protocols are not always available for published trials. To our knowledge, no study has investigated the proportion of published protocols that are associated with published results. Our objective in this study was to estimate the proportion of trial protocols that are eventually associated with published results.

## Methods

### Eligibility Criteria

This cross-sectional study was conducted as part of a larger ongoing project that aims to develop tools for assessing and improving reporting clinical trial protocols and results and followed the Strengthening the Reporting of Observational Studies in Epidemiology (STROBE) reporting guideline. For this substudy, we included open access protocols registered on ClinicalTrials.gov. We included parallel randomized clinical trials. We excluded self-identified pilot or feasibility studies as well as studies that were ongoing and studies within 1 year of the primary completion date on ClinicalTrials.gov because these trials might not have had time to publish results (eAppendix 2 in Supplement 1). This substudy was part of a larger ongoing project that was determined to be exempt by the University of Illinois Urbana-Champaign Office for the Protection of Research Subjects and by the University of North Carolina Office of Human Research Ethics Research because it involved the use of educational tests, survey procedures, interview procedures, or observation of public behavior and the information obtained was recorded in such a manner that the identity of the human participants cannot readily be ascertained.

### Search Strategy to Identify Trial Protocols

We used stratified random sampling to identify eligible protocols. First, we searched PubMed Central from January 2011 to August 2022 (eAppendix 3 and eTable 2 in Supplement 1). We downloaded citations and retained only those with a ClinicalTrials.gov identifier in the abstract or full text. We then randomly selected 500 articles from each year, yielding 6000 citations with a ClinicalTrials.gov identifier from 2011 to 2022. To identify potentially eligible protocols, we ordered these citations randomly; 3 of us (C.J.V., A.W.B., and E.M-W.) then screened the first 1500 citations in duplicate and resolved discrepancies through discussion. Thus, while our search strategy identified more records in more recent years (eg, 577 in 2011 compared with 4690 in 2021), we included comparable numbers of protocols in all years.

### Search Strategy to Identify Published Results for Included Protocols

For each included protocol, we considered results to have been published if they appeared in 1 or more reports. To record the timing of published results, we sought to identify the earliest associated publication of the main results (eAppendix 4 in Supplement 1). First, we checked ClinicalTrials.gov, which lists published results indexed automatically using the ClinicalTrials.gov identifier or added manually by the investigators. Second, if we did not find published results for an included protocol, we emailed the protocol authors to ask whether their results had been published and to request citations. Third, we attempted to identify additional published results using an automated tool that identifies candidate articles associated with studies registered on ClinicalTrials.gov.^26^ We also recorded any trial publications identified incidentally, such as those listed in the “Cited By” feature on PubMed or referenced in another publication that we reviewed for eligibility. Protocols were excluded during these steps if we learned that the trials did not match our inclusion criteria. Our search for published results started in August 2022 and ended in March 2023.

### Characteristics of the Included Trials

We obtained data about each trial registration from ClinicialTrials.gov (eTable 3 in Supplement 1). We obtained data about each protocol and results publication from PubMed, including the journal, publisher, and publication date. We obtained each journal’s 2021 impact factor from Web of Science.^27^

### Statistical Analysis

We calculated the proportion of included protocols for which we did not find published results. We then estimated the number of eligible protocols on PubMed Central without published results in each year from 2011 to 2022. Finally, we calculated a weighted estimate for the entire period based on the estimated number of protocols published in each year, and we used bootstrapping to calculate the percentile-based 95% CI for the estimate (eAppendix 5 in Supplement 1). We used the Kaplan-Meier method to draw a curve showing the cumulative probability of results publication over time.

We report descriptive characteristics of included trials. We calculated overall agreement for protocol screening. We also assessed interrater reliability using the Cohen κ coefficient,^28^ calculated using the Python package scikit-learn, version 1.2.2. All analyses were performed using Python, version 3.7 (Python Software Foundation).

## Results

### Results of the Search for Trial Protocols

From 1500 citations screened, we identified 364 potentially eligible randomized clinical trial protocols. Overall agreement between independent raters was 94%, and the Cohen κ was 0.85.

**Figure 1.**
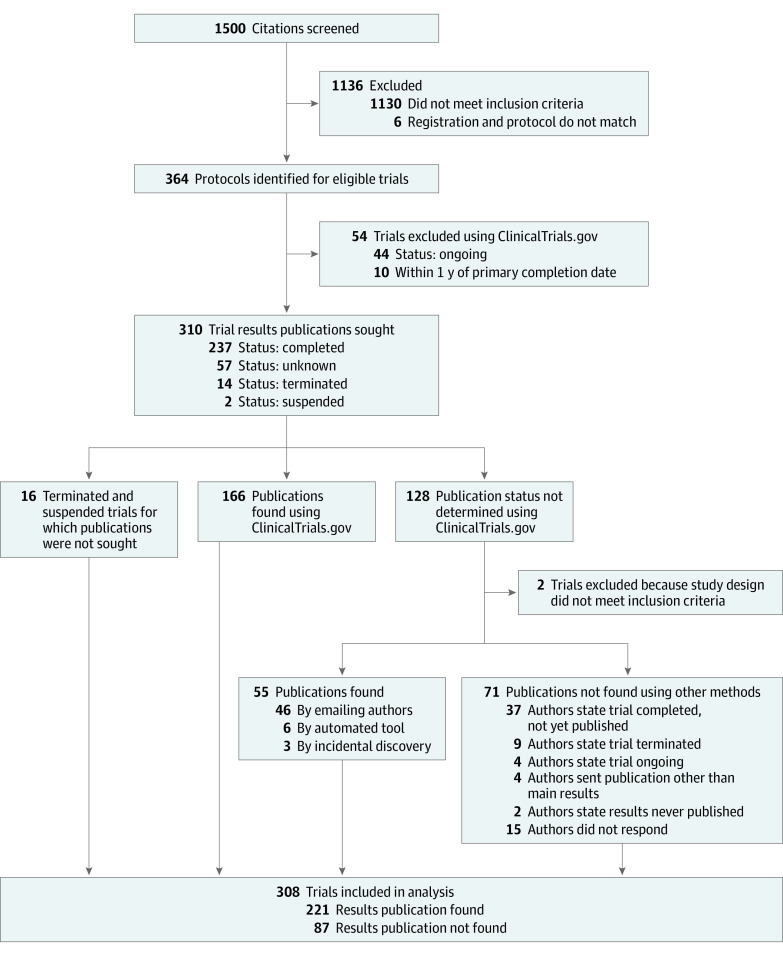
Flowchart of Article Screening to Identify Protocols and Associated Publication of Main Results

Of the 364 protocols, we excluded 54 trials using information from ClinicalTrials.gov, including 44 ongoing trials and 10 trials within 1 year of the primary completion date.^29^ After locating the published results, we also excluded 2 trials; 1 was not a complete protocol, and 1 was not truly randomized according to the reported methods. Thus, we included 308 trials in our analysis (Figure 1).

### Results of the Search for Published Results

We did not identify published results for 87 of the 308 included trials. We located publications for 166 trials using ClinicalTrials.gov, 46 by contacting authors, 6 using an automated tool, and 3 using incidental manual methods (Figure 1).

Although all 308 protocols were freely available in PubMed Central, we found a results publication in PubMed Central for only 119 of the 221 trials with published results (54%). We found 97 other publications (44%) in PubMed but not PubMed Central, and we found 5 in neither database (2%).

Authors replied by email that manuscripts were in preparation or under review for 26 and 10 trials, respectively (Table 1). Authors replied that 9 trials had been terminated and 4 were not complete. Four trials that the authors described as terminated had a status of completed on ClinicalTrials.gov; the 4 described as not yet completed by the authors were listed as unknown status on ClinicalTrials.gov. Authors confirmed nonpublication of only 2 trials. We did not receive responses for 23 trials for which we could not locate a results publication.

**Table 1.** Author Responses to Emails

Authors’ reply	No. (%) of responses (n = 126^a^)
Sent article that we confirmed was main results publication	46 (37)
Manuscripts in preparation for publication	26 (21)
No response	23 (18)
Manuscript under review	10 (8)
Trial was terminated	9 (7)
Trial not yet complete	4 (3)
Sent articles that were not main published results	4 (3)
Results never published	2 (2)
Manuscript accepted for publication but not yet published	1 (1)
Acknowledged receipt of our email but did not send publication information	1 (1)

^a^ Includes trials for which we emailed 1 or more authors and includes the 9 trials for which we found published results through the automated tool and incidental discovery.

### Characteristics of the Included Trials

Protocols were published between January 2011 and March 2022. Registrations of included trials were submitted to ClinicalTrials.gov between September 2005 and April 2021. ClinicalTrials.gov included a primary completion date for 306 trials, which were between June 2007 and February 2022 (Table 2).^29^ When we completed our data collection in March 2023, the mean (SD) time that a protocol had been published was 7.5 (3.0) years ago, with a primary completion date of 6.3 (3.0) years ago. Additional time characteristics are reported in eTable 4 in Supplement 1.

**Table 2.** Characteristics of Included Trials^a^

Characteristic	All included trials (N = 308)	Trials with published results (n = 221)	Trials without published results (n = 87)
Trial registration characteristics posted on ClinicalTrials.gov			
Trial status, No. (%)			
Completed	236 (77)	192 (87)	44 (51)
Unknown status	56 (18)	29 (13)	27 (31)
Terminated	14 (5)	0	14 (16)
Suspended	2 (1)	0	2 (2)
Actual or planned enrollment, median (IQR), No.	200 (100-501)^b^^,^^c^	232 (107-600)	154 (80-335)^b^^,^^c^
Trial phase, No. (%)			
Early phase 1	2 (1)	2 (1)	0
Phase 1	4 (1)	2 (1)	2 (2)
Phase 2	29 (9)	18 (8)	13 (12)
Phase 3	41 (13)	28 (13)	15 (14)
Phase 4	31 (10)	26 (12)	6 (7)
Not applicable	197 (64)	143 (65)	54 (62)
No information^d^	4 (1)	2 (1)	2 (2)
Trials with results posted on ClinicalTrials.gov, No. (%)	42 (14)	35 (16)	7 (8)
No. of outcomes, median (IQR)			
Primary	1 (1-1)	1 (1-1)	1 (1-1)
Secondary	5 (2-8)	5 (3-8)	5 (2-8)
Other	2.5 (1-5.25)	3 (1-5)	2 (2-6)
Sponsor classification, No. (%)			
Other	283 (92)	202 (91)	81 (93)
Other government	13 (4)	9 (4)	4 (5)
Industry	10 (3)	9 (4)	1 (1)
Federal	1 (0.3)	0	1 (1)
Not reported	1 (0.3)	1 (0.4)	0
Design, No. (%)			
Parallel assignment	286 (93)	204 (92)	82 (94)
Single group assignment^e^	9 (3)	7 (3)	2 (2)
Factorial assignment	7 (2)	6 (3)	1 (1)
Crossover assignment^f^	1 (0.3)	1 (0.4)	0
No information	5 (2)	3 (1)	2 (2)
Design purpose, No. (%)			
Treatment	163 (53)	119 (54)	44 (51)
Prevention	60 (19)	46 (21)	14 (16)
Supportive care	26 (8)	18 (8)	8 (9)
Health services research	25 (8)	19 (9)	6 (7)
Diagnostic	5 (2)	4 (2)	1 (1)
Basic science	3 (1)	2 (1)	1 (1)
Screening	2 (1)	2 (1)	0
Other	14 (5)	6 (3)	8 (9)
No information	10 (3)	5 (2)	5 (6)
Design masking, No. (%)			
None (open label)	109 (35)	76 (34)	33 (38)
Single	94 (31)	65 (29)	29 (33)
Double	40 (13)	31 (14)	9 (10)
Triple	15 (5)	11 (5)	4 (5)
Quadruple	44 (14)	34 (15)	10 (11)
No information	6 (2)	4 (2)	2 (2)
Protocol publication characteristics			
Unique journals or preprint servers	65	52	27
Unique publishers	15	12	10
Journal impact factor, 2021, median (IQR)	2.7 (2.7-4.1)	2.7 (2.7-4.0)	2.9 (2.7-4.1)
Results publication characteristics			
Unique journals	NA	145	NA
Unique publishers: results	NA	29	NA
Journal impact factor, 2021, median (IQR)	NA	8.5 (4.7-22.1)	NA

Abbreviation: NA, not applicable.

^a^ For a glossary of terms, see ClinicalTrials.gov.^29^

^b^ Trial NCT01367405 reported an enrollment of 1 participant before termination, and NCT01613183 reported an enrollment of 2 participants.

^c^ Trial NCT03158181 reported an enrollment of 20 959 participants and an observational design, but the associated protocol (PMC6528209) describes a cluster-randomized trial, so we included it in our analysis.

^d^ These registrations described observational designs, but their associated protocols described clinical trials, so we included them in our analysis.

^e^ Some trials listed single group assignment as the intervention model, but their protocols described random assignment to multiple arms. These trials were included in our analysis.

^f^ Trial registration states crossover, but the protocol describes a parallel design, so the trial was included.

**Figure 2.**
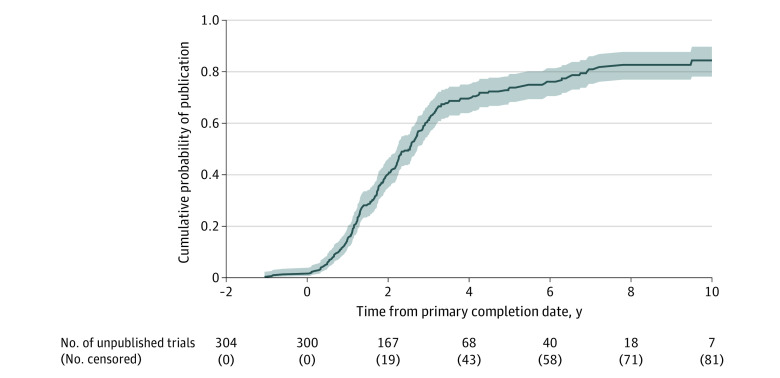
Cumulative Probability of Results Being Published Results of some trials were published prior to the estimated or actual primary completion date. The x-axis was cropped at 10 years after the primary completion date because few trials were followed up for more than 10 years. The shaded area indicates the 95% CI.

Results were published between March 2012 and March 2023. Published results appeared a mean (SD) of 3.4 (2.0) years after publication of its associated protocol. For the 306 trials with primary completion dates, the first published results appeared a mean (SD) of 2.2 (1.8) years after the primary completion dates. With the Kaplan-Meier method, 50% of trials had published results by 2.5 years after the primary completion date (Figure 2). The trial with the longest time “at risk” published results 10.7 years after protocol publication and 13.5 years after the primary completion date (Figure 2; eFigure in Supplement 1).

Whereas 286 protocols reported the ClinicalTrials.gov identifier in the abstract, only 69 published results included the registration number in the abstract following the 2010 Consolidated Standards of Reporting Trials (CONSORT) reporting guideline.^30^ Trial status was completed for most trials (236 [77%]), but 56 trials (18%) had unknown status, which indicates that their records had passed the primary completion date but investigators had not verified the records for at least 2 years (Table 2^29^). Most of the included trials (197 [64%]) described the trial phase as not applicable, a status sometimes used to describe medical device trials and, in our sample, used commonly to describe trials that did not evaluate regulated medical products. Only 10 trials (3%) were classified as sponsored by industry. The mean numbers of outcomes were as follows: primary, 1.5 (range, 1-18); secondary, 6.3 (range 1-34); and other, 4.4 (range 1-23). Few of the included trials (42 [14%]) posted results on ClinicalTrials.gov; 7 of the 87 trials without published results (8%) posted results but had not published them. Trials with published results had a higher proportion of completed rather than unknown status and were larger.

Protocols were published in 65 different journals or preprint servers associated with 15 different publishers (eTables 5 and 6 in Supplement 1), and the median impact factor was 2.7 (IQR, 2.7-4.1). By comparison, results were published in 145 different journals associated with 29 different publishers, and the median impact factor was 8.5 (IQR, 4.7-22.1) (eTables 7 and 8 in Supplement 1).

### Estimating the Number of Published Trials

**Figure 3.**
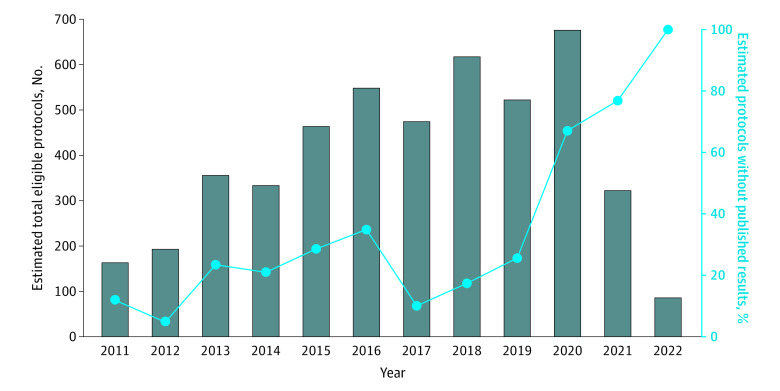
Estimated Number of Eligible Protocols on PubMed Central and Estimated Percentage of Protocols Without Published Results on PubMed Central

Using a weighted mean, we estimated that 4754 (95% CI, 4296-5226) protocols matching our inclusion criteria were published and indexed in PubMed Central from 2011 to 2022 and that 1708 (36%; 95% CI, 31%-41%) protocols did not have associated published results (Figure 3; eTable 9 in Supplement 1). In our sample, trials with published results tended to be older than trials with unpublished results. The mean (SD) time that results were published was 3.4 (2.0) years after the associated protocol. We also identified a steep increase in the proportion of trials without published results beginning in 2020 (Figure 3; eTable 9 in Supplement 1).

We conducted a post hoc sensitivity analysis restricted to protocols published before 2020. In our sample, the proportion of protocols without published results for the years 2011 to 2019 was 23% (63 of 276) compared with 28% (87 of 308) of protocols overall. We estimated that 3670 (95% CI, 3310-4032) eligible protocols were published between 2011 and 2019 and that 25% (95% CI, 20%-30%) did not have associated published results (eTable 9 in Supplement 1), somewhat lower than our overall weighted estimate that 36% of trials did not have published results.

## Discussion

Of 308 included clinical trial protocols, 87 had not published their main results. In our primary weighted analysis, we estimated that 36% of trials with protocols published since 2011 had not published their results. To our knowledge, this is the first study to quantify research waste associated with nonpublication of main results from published trial protocols.

This study highlights deficiencies in the reporting of clinical trials. For example, we were unable to determine the status of many unpublished trials because information on ClinicalTrials.gov was often outdated and because some investigators did not respond to requests for information. Few trials posted results on ClinicalTrials.gov. We also found that information in registries and protocols was incorrect for some studies. Although the 2010 CONSORT guideline states that the registration number should be included in the abstract,^31^ many published results were difficult to associate with corresponding protocols because they did not include trial registration numbers in the abstracts. As in another recent study,^32^ we conclude that consistent inclusion of the registration number in abstracts would facilitate tracking and linking of protocols and published results.

Despite nonpublication of many trial results, the benefits of protocol publication could outweigh wasted publication charges and wasted investigator, reviewer, and editorial time. Even in the absence of published results, some protocols might be useful to the authors and to others. More likely, publishing protocols might reduce research waste from biased or unusable trial results. Because trials can be costly and impactful, reducing waste from only a few trials could avoid millions of dollars in wasted research or health care spending. By comparison, if approximately 2000 protocols each cost approximately $3000 (roughly the cost of an article processing fee plus time to review the manuscript), then failure to disseminate the results after publishing trial protocols indexed in PubMed Central might account for $6 million in research waste since 2011. The greatest harms stemming from nonpublication of trials probably arise from the loss of scientific information and from the violations of ethical commitments to participants who enrolled in trials whose results were not published. On the other hand, authors could achieve many of the benefits of publishing protocols in journals by posting them on preprint servers without charge.

### Limitations

Our study has some limitations. First, we included and characterized trials using information that investigators provided to ClinicalTrials.gov, some of which was outdated or incorrect. Second, we might not have located the main published results for all trials. To include complete and accurate information about trial status and publication status, we conducted comprehensive searches of PubMed and contacted investigators directly. Nonetheless, we neither identified published results nor received a response from the investigators of 25 trials. Although some recent trials might be published in the future, our conclusions would not be meaningfully different based on a sensitivity analysis restricted to older trials that had ample time to publish their results. Trials with protocols published from 2019 to 2022 might have been affected by COVID-19; the pandemic’s effect on the probability of publication and time to publication is not yet known. Third, this study was part of a larger investigation and was not registered separately.

Our results are most generalizable to behavioral intervention and prevention research. Few trials of drugs and biologics were identified from our search. Most trials were investigator initiated rather than industry sponsored. For these reasons, our results might differ from studies examining the publication of trials in ClinicalTrials.gov and other registries, which include many trials of regulated products sponsored by drug and device manufacturers. Our sample was also limited to protocols published on PubMed Central that included a ClinicalTrials.gov identifier, so our results might not be generalizable to protocols published in journals indexed in other databases or in other registries, or to trials that were not registered.

## Conclusions

This cross-sectional study suggests that many published trial protocols were not associated with subsequent published results. Moreover, many trials did not adhere to best practices for registering and reporting their status and results. Despite these deficiencies, the benefits of publishing trial protocols probably outweigh the associated research waste. Many of those benefits could be achieved by posting protocols on preprint servers without charge.

## Notes

Supplement 1. **eAppendix 1.** Background **eTable 1.** Examples of Costs to Publish Open Access Trial Protocols **eAppendix 2.** Inclusion and Exclusion Criteria for RCT Protocol Screening **eAppendix 3.** Search String Development **eTable 2.** PubMed Search String Used to Identify RCT Protocols **eAppendix 4.** Screening **eAppendix 5.** Supplemental Results **eTable 3.** ClinicalTrials.gov Fields Collected **eTable 4.** Trial Time Characteristics of Included Trials **eTable 5.** Frequency of Included Protocol Publications in Journals and Preprint Servers **eTable 6.** Frequency of Included Protocol Publications by Publisher **eTable 7.** Frequency of Included Results Publications by Journal **eTable 8.** Frequency of Included Results Publications by Publisher **eTable 9.** Estimated Number of Eligible Protocols on PubMed Central Without Published Results **eFigure.** Cumulative Probability of Results Being Published After Estimated or Actual Primary Completion Date Listed on ClinicalTrials.gov, From Protocol Publication Date 2011 to 2019
eReferences.

Supplement 2.
Data Sharing Statement
