# Supplementary material for: Publication of Results of Registered Trials With Published Study Protocols, 2011-2022
Source: JAMA Netw Open. 2024 Jan 8;7(1):e2350688. doi: 10.1001/jamanetworkopen.2023.50688 (PMC10774993; doi:10.1001/jamanetworkopen.2023.50688)
Supplement: Supplement 1. — eAppendix 1. Background eTable 1. Examples of Costs to Publish Open Access Trial Protocols eAppendix 2. Inclusion and Exclusion Criteria for RCT Protocol Screening eAppendix 3. Search String Development eTable 2. PubMed Search String Used to Identify RCT Protocols eAppendix 4. Screening eAppendix 5. Supplemental Results eTable 3. ClinicalTrials.gov Fields Collected eTable 4. Trial Time Characteristics of Included Trials eTable 5. Frequency of Included Protocol Publications in Journals and Preprint Servers eTable 6. Frequency of Included Protocol Publications by Publisher eTable 7. Frequency of Included Results Publications by Journal eTable 8. Frequency of Included Results Publications by Publisher eTable 9. Estimated Number of Eligible Protocols on PubMed Central Without Published Results eFigure. Cumulative Probability of Results Being Published After Estimated or Actual Primary Completion Date Listed on ClinicalTrials.gov, From Protocol Publication Date 2011 to 2019 eReferences. [file jamanetwopen-e2350688-s001.pdf]

Supplementary Online Content

Vorland CJ, Brown AW, Kilicoglu H, Ying X, Mayo-Wilson E. Publication of results of registered trials with published study protocols, 2011-2022. *JAMA Netw Open.* 2024;7(1):e2350688. doi:10.1001/jamanetworkopen.2023.50688

eAppendix 1. Background
eTable 1. Examples of Costs to Publish Open Access Trial Protocols
eAppendix 2. Inclusion and Exclusion Criteria for RCT Protocol Screening
eAppendix 3. Search String Development
eTable 2. PubMed Search String Used to Identify RCT Protocols
eAppendix 4. Screening
eAppendix 5. Supplemental Results
eTable 3. ClinicalTrials.gov Fields Collected
eTable 4. Trial Time Characteristics of Included Trials
eTable 5. Frequency of Included Protocol Publications in Journals and Preprint Servers
eTable 6. Frequency of Included Protocol Publications by Publisher
eTable 7. Frequency of Included Results Publications by Journal
eTable 8. Frequency of Included Results Publications by Publisher
eTable 9. Estimated Number of Eligible Protocols on PubMed Central Without Published Results
eFigure. Cumulative Probability of Results Being Published After Estimated or Actual Primary Completion Date Listed on ClinicalTrials.gov, From Protocol Publication Date 2011 to 2019
eReferences.

This supplementary material has been provided by the authors to give readers additional information about their work.

eAppendix 1. Background

eTable 1. Examples of Costs to Publish Open Access Trial Protocols

BMC Public Health	$2,990[1]
BMJ Open (a BMJ journal)	$2,645[2]
Clinical Trials [a]	$3,700[3]
Contemporary Clinical Trials [a]	$2,900[4]
JMIR Research Protocols	$1,900[6]
Pilot and Feasibility Studies (a BMC journal)	$2,290[1]
Trials (a BMC journal)	$2,390[7]

[a] Journal offers a non-open access publication option without an article-processing charge.

© 2024 Vorland CJ et al. *JAMA Network Open.*

eAppendix 2. Inclusion and Exclusion Criteria for RCT Protocol Screening

Articles were included if:

I. They used the word “protocol” or similar words/phrases such as “study design and methods” in the full text that indicated a plan for conducting a trial.

II. They stated or implied that random assignment was used:
   A. The title or abstract used the word “random” or “randomized” to describe assignment, or
   B. The title or abstract used a word such as “experiment” or “by chance” that implied the investigators both (1) will assign participants to groups and (2) that assignment will be random.

III. The study described was a “clinical trial”. Clinical trials:
   A. Involve humans (not animals)
   B. Evaluate the effect of an intervention
   C. Measure effects on health (e.g., death, disease status, symptoms) or health behaviors (e.g., smoking)

Articles were excluded if:

IV. They included trial results, including baseline data and interim analyses.

V. They did not describe overall plans for a trial, including reports limited to statistical analysis plans.

VI. They described plans for multiple trials (>1).

VII. They described only sub-studies (e.g., secondary hypotheses) within larger trials.

VIII. They described a design of crossover, stepped-wedge, and other non-parallel designs.

IX. They described a pilot or feasibility trial as defined by the authors. We defined pilot and feasibility studies as trials designed to determine whether and how to conduct (larger) clinical trials.

X. They described baseline characteristics and interim analyses.

© 2024 Vorland CJ et al. *JAMA Network Open.*

eAppendix 3. Search String Development

We developed a PubMed search string to identify open access protocols of RCTs: (1) using the Cochrane Highly Sensitive Search Strategy RCT filter [8] (2) using a modification of the protocol search string developed by Moraes Silva et al. [9] (3) restricting the publication date from 2011 to 2022, and (4) restricting articles to the PubMed Central 'Open Access' or 'Author Manuscript' subsets. Below is the search string, run on 08/10/2022:

eTable 2. PubMed Search String Used to Identify RCT Protocols

Search number	Query	Results
18	#16 AND #17	108,713
17	"2011"[Date - Publication] : "3000"[Date - Publication]	13,687,588
16	#13 AND (#14 OR #15)	120,089
15	author manuscript[filter]	884,677
14	pubmed pmc open access[filter]	4,341,427
13	#11 AND #12	608,353
12	protocol*[tiab] OR study design [tiab] OR trial design[tiab] OR research design[tiab] OR "design and methods"[tiab] OR "design and rationale"[tiab] OR "rationale and design"[tiab] OR Research Design[mh] OR clinical protocols[mh] OR clinical trial protocol[pt]	1,397,911
11	#9 NOT #10	4,798,533
10	animals [mh] NOT humans [mh]	5,032,865
9	#1 OR #2 OR #3 OR #4 OR #5 OR #6 OR #7 OR #8	5,503,020
8	"groups"[Title/Abstract]	2,422,315
7	"trial"[Title/Abstract]	719,658
6	"randomly"[Title/Abstract]	389,305
5	"drug therapy"[MeSH Subheading]	2,516,309
4	"placebo"[Title/Abstract]	237,074
3	"randomized"[Title/Abstract]	622,032
2	"controlled clinical trial"[Publication Type]	665,491
1	"randomized controlled trial"[Publication Type]	575,417

© 2024 Vorland CJ et al. *JAMA Network Open.*

eAppendix 4. Screening

ClinicalTrials.gov. Publications on each ClinicalTrials.gov registration listed under the 'More information' section were manually checked for articles. Candidate articles were opened and assessed, and main results publications were recorded if identified. Results posted to ClinicalTrials.gov were not counted as results publications.

Emailing authors. Author responses to emails requesting main results publications were classified by CJV and EM. Authors who did not reply were emailed again one week after the first email was sent. If emails bounced, alternative email addresses were manually obtained from protocols or searching the web and emails resent until no automated reply was received.

Automated tool that identifies candidate results articles from registrations on ClinicalTrials.gov: For protocols with no identified results publications from manual screening of ClinicalTrials.gov and no response from emailing authors, we used an automated tool[10] to identify candidate articles in PubMed. Candidate articles were screened to identify main results publications after excluding protocols. During this process, if no results publication was identified, we also opened the protocol in PubMed and checked PubMed's similar articles and cited by lists to identify results publications.

© 2024 Vorland CJ et al. *JAMA Network Open.*

eAppendix 5. Supplemental Results

Because we employed stratified random sampling, and because the likelihood of finding published results might vary across strata (i.e., years), we incorporated weighting in our calculation to ensure the sample estimates reflect the population parameters (see Table S9). For each year, we started by determining the proportion of eligible protocols in our sample. This proportion was then multiplied by the number of articles found via our PMC search to project the total number of eligible protocols on PMC. We then computed the annual proportion of eligible protocols lacking published results. To account for the fluctuation in the number of eligible protocols across different years, we applied weighting to each year's proportion based on the respective number of eligible protocols. This approach allowed us to calculate the overall proportion of eligible protocols without result publications over the entire study period. The formula used for this weighted calculation is as follows:

$$Weighted\ Proportion = \frac{\sum_{year=2011}^{2022} (Proportion_{year} * Weight_{year})}{\sum_{year=2011}^{2022} (Weight_{year})}$$

For the estimates, we calculated the percentile-based 95% confidence intervals using 10,000 bootstrapping replications. The R code for the calculations is available on OSF (https://doi.org/10.17605/OSF.IO/RKQ3N).

© 2024 Vorland CJ et al. *JAMA Network Open.*

eTable 3. ClinicalTrials.gov Fields Collected

**Concept**	**Field**
Trial status	overall recruitment status<br>why study stopped
Design characteristics	interventional study model<br>primary purpose<br>masking
Enrollment	"actual" or "anticipated"
Outcomes	primary<br>secondary<br>other
Other characteristics	phase<br>results first submitted
Registration timelines	first submitted<br>first posted<br>last update posted<br>record verification date<br>primary completion date<br>study completion date

© 2024 Vorland CJ et al. *JAMA Network Open.*

eTable 4. Trial Time Characteristics of Included Trials

	All Included Trials (n=308)	Trials With Published Results (n=221)	Trials Without Published Results (n=87)
**Time Characteristics (median, IQR)***			
**Date of registration submission**	Mar 2014 (Aug 2011 to Mar 2016)	May 2013 (May 2011 to Sep 2015)	May 2015 (Nov 2012 to May 2017)
**Date of registration first posted**	Mar 2014 (Sep 2011 to Mar 2016)	May 2013 (May 2011 to Sep 2015)	Jun 2015 (Dec 2012 to Jun 2017)
**Date last verified**	Apr 2018 (Nov 2015 to Apr 2020)	Oct 2017 (Nov 2015 to Oct 2019)	Jun 2019 (Apr 2016 to Apr 2021)
**Date last updated**	Jun 2018 (Feb 2016 to May 2020)	Jan 2018 (Dec 2015 to Jan 2020)	Apr 2020 (Sep 2016 to May 2021)
**Date of primary completion (estimated or actual)**	Dec 2016 (Jul 2014 to Jun 2019)	Apr 2016 (Feb 2014 to Oct 2018)	Feb 2019 (May 2016 to Dec 2020)
**Date of study completion (estimated or actual)**	Dec 2019 (Nov 2018 to Feb 2021)	Jun 2019 (Mar 2018 to Jun 2020)	Dec 2020 (Mar 2020 to Nov 2021)
**Date of protocol publication**	Jul 2015 (Feb 2013 to Feb 2018)	Oct 2014 (Aug 2012 to Aug 2017)	Oct 2016 (May 2014 to Apr 2020)
**Date of results publication**	-	Dec 2018 (Jun 2016 to Oct 2020)	-

*If dates extracted from ClinicalTrials.gov did not contain a day, the first of the month was used.

eTable 5. Frequency of Included Protocol Publications in Journals and Preprint Servers

Trials	108
BMJ Open	34
BMC Public Health	21
JMIR Research Protocols	16
BMC Cancer	12
BMC Musculoskeletal Disorders	10
BMC Psychiatry	9
BMC Geriatrics	5
BMC Health Services Research	5
BMC Neurology	5
BMC Pregnancy and Childbirth	5
Contemporary Clinical Trials Communications	5
Implementation Science	5
Medicine	4
BMC Infectious Diseases	3
BMC Pediatrics	3
BMC Women's Health	3
Contemporary Clinical Trials	3
BMC Complementary and Alternative Medicine	2
BMC Emergency Medicine	2
BMC Nephrology	2
BMC Psychology	2
Evidence-based Complementary and Alternative Medicine	2
Acta Diabetologica	1
Addiction Science & Clinical Practice	1
BMC Cardiovascular Disorders	1
BMC Dermatology	1
BMC Family Practice	1

© 2024 Vorland CJ et al. *JAMA Network Open.*

BMC Gastroenterology	1
BMC Medical Education	1
BMC Palliative Care	1
BMC Pulmonary Medicine	1
BMC Surgery	1
BMJ Global Health	1
BMJ Open Respiratory Research	1
Bone & Joint Research	1
Cardiovascular Diabetology	1
Cardiovascular Drugs and Therapy	1
Clinical Breast Cancer	1
Clinical Cardiology	1
Colorectal Disease	1
Contemporary clinical trials	1
Esc Heart Failure	1
European Eating Disorders Review	1
European Journal of Heart Failure	1
F1000Research	1
Fertility and Sterility	1
Frontiers in Medicine	1
Global Mental Health	1
Health & Justice	1
International Journal of Chronic Obstructive Pulmonary Disease	1
International Journal of Environmental Research and Public Health	1
Journal of Cardiac Failure	1
Journal of Cardiovascular Translational Research	1
Journal of Neural Transmission	1
Journal of Thrombosis and Thrombolysis	1
Journal of the American Heart Association	1

© 2024 Vorland CJ et al. *JAMA Network Open.*

Methods and Protocols	1
Nutrition Journal	1
PLoS ONE	1
Respiratory Research	1
Scandinavian Journal of Trauma, Resuscitation and Emergency Medicine	1
The Journals of Gerontology Series A: Biological Sciences and Medical Sciences	1
The Open Nursing Journal	1
medRxiv	1

© 2024 Vorland CJ et al. *JAMA Network Open.*

eTable 6. Frequency of Included Protocol Publications by Publisher

Springer Nature	221
BMJ Publishing Group	36
JMIR Publications	16
Elsevier	12
Wiley	7
Wolters Kluwer	4
MDPI	2
Oxford University Press	2
Taylor and Francis	2
Bentham Open	1
British Editorial Society of Bone and Joint Surgery	1
Cambridge University Press	1
Cold Spring Harbor Laboratory	1
Frontiers Media S.A.	1
Public Library of Science	1

To the extent of our knowledge, we identified major publishers at present ownership structures. For example, articles published in BioMed Central were classified under Springer Nature; Hindawi was classified under Wiley; Lippincott Williams & Wilkins under Wolters Kluwer; Dove Press under Taylor and Francis.

© 2024 Vorland CJ et al. *JAMA Network Open.*

eTable 7. Frequency of Included Results Publications by Journal

PLoS Medicine	7
JAMA	6
Lancet	6
The New England Journal of Medicine	6
PLoS ONE	5
BMC Medicine	4
Circulation	4
Diabetes Care	4
JAMA Internal Medicine	4
American Journal of Obstetrics and Gynecology	3
BMC Psychiatry	3
BMJ Open	3
European Heart Journal	3
JAMA Network Open	3
Journal of Clinical Oncology	3
The BMJ	3
Age and Ageing	2
American Journal of Preventive Medicine	2
Annals of Surgery	2
BMC Public Health	2
Clinical Infectious Diseases	2
Frontiers in Pharmacology	2
Gerontology	2
JMIR mHealth and uHealth	2
Journal of Clinical Medicine	2
Journal of Consulting and Clinical Psychology	2
Journal of General Internal Medicine	2
Journal of Medical Internet Research	2

© 2024 Vorland CJ et al. *JAMA Network Open.*

Journal of Physiotherapy	2
Nutrients	2
Nutritional Neuroscience	2
PLoS Neglected Tropical Diseases	2
Palliative Medicine	2
Pediatric Obesity	2
Pediatrics	2
Psycho-oncology	2
The British Journal of Surgery	2
The Lancet Global Health	2
The Lancet Respiratory Medicine	2
AIDS Research and Therapy	1
Acupuncture in Medicine	1
Addiction	1
American Journal of Respiratory and Critical Care Medicine	1
American Journal of Transplantation	1
Anesthesiology	1
Annals of Behavioral Medicine	1
Annals of Internal Medicine	1
Annals of Neurology	1
Annals of Oncology	1
Arthritis & Rheumatology	1
Arthritis Care & Research	1
BJOG	1
BMC Complementary and Alternative Medicine	1
BMC Health Services Research	1
BMC Nephrology	1
BMC Pediatrics	1
BMC Research Notes	1

© 2024 Vorland CJ et al. *JAMA Network Open*.

Beneficial Microbes	1
Brain Injury	1
Cancer	1
Cardiovascular Revascularization Medicine	1
Child and Adolescent Psychiatry and Mental Health	1
Childhood Obesity	1
Chinese Journal of Integrative Medicine	1
Circulation Research	1
Clinical Biomechanics	1
Clinical Microbiology and Infection	1
Clinical Nutrition	1
Clinical Nutrition ESPEN	1
Clinical Orthopaedics and Related Research	1
Clinical Rehabilitation	1
Contact Dermatitis	1
Critical Care	1
Drug and Alcohol Dependence	1
EClinicalMedicine	1
EP Europace	1
Ear and Hearing	1
European Journal of Cancer	1
European Journal of Midwifery	1
Family & Community Health	1
Frontiers in Neurology	1
Frontiers in Psychiatry	1
Frontiers in Psychology	1
Heart	1
Injury	1
International Journal of Environmental Research and Public Health	1

International Journal of Radiation Oncology, Biology, Physics	1
JACC Cardiovascular Interventions	1
JACC Heart failure	1
JAMA Neurology	1
JAMA Pediatrics	1
JAMA Psychiatry	1
JBJS Open Access	1
JCPP Advances	1
JMIR Public Health and Surveillance	1
Journal of Acquired Immune Deficiency Syndromes	1
Journal of Affective Disorders	1
Journal of Child and Family Studies	1
Journal of Clinical Anesthesia	1
Journal of Clinical Periodontology	1
Journal of Ethnopharmacology	1
Journal of Gastrointestinal Surgery	1
Journal of Human Lactation	1
Journal of Korean Medical Science	1
Journal of Ovarian Research	1
Journal of Pediatric Gastroenterology and Nutrition	1
Journal of Psychosomatic Research	1
Journal of Substance Abuse Treatment	1
Journal of the American Heart Association	1
Journal of the American Medical Directors Association	1
Journal of the American Society of Nephrology	1
Malaria Journal	1
Manual Therapy	1
Maternal & Child Nutrition	1
NPJ Digital Medicine	1

© 2024 Vorland CJ et al. *JAMA Network Open*.

Nursing Research	1
Pain	1
Palliative & Supportive Care	1
Peritoneal Dialysis International	1
Pharmaceutics	1
Physical Therapy	1
Reproductive Health	1
Respiratory Medicine	1
Scandinavian Journal of Medicine & Science in Sports	1
Sports Health	1
Stroke	1
The American Journal of Clinical Nutrition	1
The American Journal of Geriatric Psychiatry	1
The American Journal of Psychiatry	1
The American Psychologist	1
The European Respiratory Journal	1
The International Journal of Behavioral Nutrition and Physical Activity	1
The Journal of Asthma	1
The Journal of Clinical Psychiatry	1
The Journal of Thoracic and Cardiovascular Surgery	1
The Journal of Urology	1
The Lancet Diabetes & Endocrinology	1
The Lancet Gastroenterology & Hepatology	1
The Lancet HIV	1
The Lancet Infectious Diseases	1
The Lancet Neurology	1
The Lancet Oncology	1
The Lancet Psychiatry	1
The Pediatric Infectious Disease Journal	1

© 2024 Vorland CJ et al. *JAMA Network Open*.

Therapeutic Advances in Neurological Disorders	1

© 2024 Vorland CJ et al. *JAMA Network Open*.

eTable 8. Frequency of Included Results Publications by Publisher

Elsevier	50
Springer Nature	25
American Medical Association	17
Wolters Kluwer	16
Public Library of Science	14
Wiley	14
Oxford University Press	12
BMJ Publishing Group	8
SAGE Publications	7
MDPI	6
Massachusetts Medical Society	6
American Heart Association	5
Frontiers Media S.A.	5
JMIR Publications	5
Taylor and Francis	4
American Diabetes Association	3
American Psychological Association	3
American Society of Clinical Oncology	3
American Academy of Pediatrics	2
Karger	2
American College of Physicians.	1
American Psychiatric Association	1
American Thoracic Society	1
Cambridge University Press	1
European Publishing	1
European Respiratory Society	1
Korean Academy of Medical Sciences	1
Physicians Postgraduate Press	1

© 2024 Vorland CJ et al. *JAMA Network Open.*

Wageningen Academic	1

To the extent of our knowledge, we identified major publishers at present ownership structures. For example, articles published in BioMed Central were classified under Springer Nature; Hindawi was classified under Wiley; Lippincott Williams & Wilkins under Wolters Kluwer; Dove Press under Taylor and Francis.

© 2024 Vorland CJ et al. *JAMA Network Open*.

eTable 9. Estimated Number of Eligible Protocols on PubMed Central Without Published Results

Year of Protocol Publication	Number of records on PMC from our search that contained a ClinicalTrials.gov identifier (N)[a]	Number of records screened (Ns)	Number of eligible protocols we identified and included from screening (Ne)	Number of results publications we found (Np)	Proportion of eligible protocols from all screened articles (Pe=Ne/Ns)	Proportion of eligible protocols without published results (Pnp=1-Np/Ne)	Estimated number of eligible protocols on PMC (Weight=Pe* N)	Estimated number of eligible protocols on PMC without published results (Pnp*Weight)
**2011**	577	138	39	33	0.283	0.154	163	25
**2012**	694	126	35	32	0.278	0.086	193	17
**2013**	1140	109	34	25	0.312	0.265	356	94
**2014**	1414	123	29	22	0.236	0.241	333	80
**2015**	1721	130	35	24	0.269	0.314	463	146
**2016**	2107	123	32	20	0.260	0.375	548	206
**2017**	2501	116	22	19	0.190	0.136	474	65
**2018**	2746	129	29	23	0.225	0.207	617	128
**2019**	3307	133	21	15	0.158	0.286	522	149
**2020**	4340	122	19	6	0.156	0.684	676	462
**2021**	4690	131	9	2	0.069	0.778	322	251
**2022**	2570	120	4	0	0.033	1.000	86	86
**Total (95%)**	27807	1500	308	221	-	-	4754 (4296, 5226)	**1708 (1393, 2042)**

© 2024 Vorland CJ et al. *JAMA Network Open.*

								**36% (31%, 41%)**
**Sensitivity analysis (95%)[b]**	16207	1127	276		-		3670 (3310, 4032)	**909 (714, 1111)**<br>**25% (20%, 30%)**

[a] We randomly sampled 500 records from each year for screening, and randomly screened 1500 records from that list. Thus, the "number of records screened" and the "number of eligible protocols identified and included from screening" are comparable across most years. Estimates are weighted by the total number of protocols found in each year.

[b] We conducted a sensitivity analysis limited to protocols published from 2011 to 2019.

© 2024 Vorland CJ et al. *JAMA Network Open.*

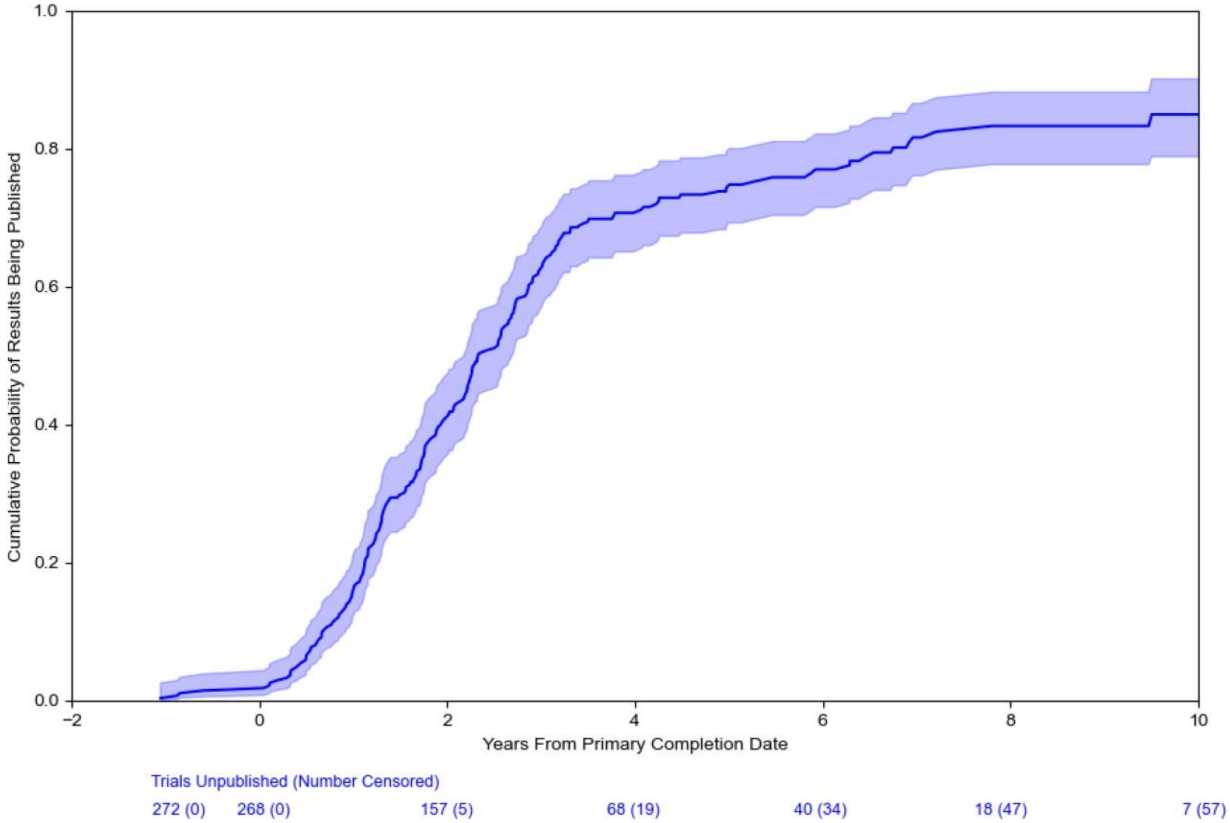

eFigure. Cumulative Probability of Results Being Published After Estimated or Actual Primary Completion Date Listed on ClinicalTrials.gov, From Protocol Publication Date 2011 to 2019. Blue line: cumulative probability of publication over time; light blue shading: 95% confidence interval; blue text: cumulative results published (cumulative number censored). Results publications of some trials were published prior to the estimated or actual primary completion date. The x-axis was cropped at 10 years after the primary completion date because few trials were followed for more than 10 years.

© 2024 Vorland CJ et al. *JAMA Network Open.*

eReferences.

1. Springer Nature. Article-processing charges. Accessed 07/21/2023, https://bmcpublichealth.biomedcentral.com/submission-guidelines/fees-and-funding
2. Authors. BMJ Publishing Group Ltd. Accessed 04/30/2023, https://bmjopen.bmj.com/pages/authors
3. Sage Choice. Accessed 07/21/2023, https://us.sagepub.com/en-us/nam/sage-choice
4. Elsevier. Open access options. Accessed 07/21/2023, https://www.elsevier.com/journals/contemporary-clinical-trials/1551-7144/open-access-options
5. JAMA Network. For Authors. Accessed 07/21/2023, https://jamanetwork.com/journals/jamanetworkopen/pages/for-authors
6. JMIR Publications. Article Processing Fees. Accessed 07/21/2023, https://www.researchprotocols.org/fees/article-processing-fees
7. Article-processing charges. Springer Nature. Accessed 04/30/2023, https://trialsjournal.biomedcentral.com/submission-guidelines/fees-and-funding
8. Cochrane Highly Sensitive Search Strategy for identifying randomized trials in MEDLINE: sensitivity-maximizing version (2008 revision); PubMed format. In: Green JPHaS, ed. *Cochrane Handbook for Systematic Reviews of Interventions*.
9. Silva FM, Adegboye ARA, Curioni C, et al. Protocol for a meta-research study of protocols for diet or nutrition-related trials published in indexed journals: general aspects of study design, rationale and reporting limitations. *BMJ open*. 2022;12(12):e064744.
10. Smalheiser NR, Holt AW. A web-based tool for automatically linking clinical trials to their publications. *Journal of the American Medical Informatics Association*. 2022;29(5):822-830.

© 2024 Vorland CJ et al. *JAMA Network Open*.
