# Supplementary material for: Publication of Results of Registered Trials With Published Study Protocols, 2011-2022
Source: JAMA Netw Open. 2024 Jan 8;7(1):e2350688. doi: 10.1001/jamanetworkopen.2023.50688 (PMC10774993; doi:10.1001/jamanetworkopen.2023.50688)
Supplement: Supplement 2. — Data Sharing Statement [file jamanetwopen-e2350688-s002.pdf]

Data Sharing Statement

Vorland. Publication of Results of Registered Trials With Published Study Protocols, 2011-2022. *JAMA Netw Open*. Published January 08, 2024.
doi:10.1001/jamanetworkopen.2023.50688

Data
Data available: Yes
Data types: Data (not involving human participants), Data dictionary
How to access data: https://doi.org/10.17605/OSF.IO/RKQ3N
When available: With publication

Supporting Documents
Document types: Statistical/analytic code
How to access documents: https://doi.org/10.17605/OSF.IO/RKQ3N
When available: With publication

Additional Information
Who can access the data: Data are publicly available.
Types of analyses: For any purpose.
Mechanisms of data availability: Data are publicly available.
